# Ergogenic value of oxygen supplementation in chronic obstructive pulmonary disease

**DOI:** 10.1007/s11739-022-03037-2

**Published:** 2022-07-12

**Authors:** Dimitrios Megaritis, Peter D. Wagner, Ioannis Vogiatzis

**Affiliations:** 1 Department of Sport, Exercise and Rehabilitation, Faculty of Health and Life Sciences, Northumbria University, Tyne and Wear, Newcastle upon Tyne, UK; 2 Department of Medicine, University of California, San Diego, CA USA

**Keywords:** Oxygen supplementation, COPD, Exercise tolerance

## Abstract

Patients with COPD exhibit limited exercise endurance time compared to healthy age-matched individuals. Oxygen supplementation is often applied to improve endurance time during pulmonary rehabilitation in patients with COPD and thus a comprehensive understanding of the mechanisms leading to improved endurance is desirable. This review analyses data from two studies by our research group investigating the effect of oxygen supplementation on cerebrovascular, systemic, respiratory and locomotor muscle oxygen availability on the same cohort of individuals with advanced COPD, and the mechanisms associated with improved endurance time in hyperoxia, which was essentially doubled (at the same power output). In hyperoxia at isotime (the time at which patients became exhausted in normoxia) exercise was associated with greater respiratory and locomotor muscle (but not frontal cortex) oxygen delivery (despite lower cardiac output), lower lactate concentration and less tachypnoea. Frontal cortex oxygen saturation was higher, and respiratory drive lower. Hence, improved endurance in hyperoxia appears to be facilitated by several factors: increased oxygen availability to the respiratory and locomotor muscles, less metabolic acidosis, and lower respiratory drive. At exhaustion in both normoxia and hyperoxia, only cardiac output and breathing pattern were not different between conditions. However, minute ventilation in hyperoxia exceeded the critical level of ventilatory constraints (V_E_/MVV > 75–80%). Lactate remained lower and respiratory and locomotor muscle oxygen delivery greater in hyperoxia, suggesting greater muscle oxygen availability improving muscle function. Taken together, these findings suggest that central haemodynamic and ventilatory limitations and not contracting muscle conditions dictate endurance time in COPD during exercise in hyperoxia.

## Introduction

**Fig. 1:**
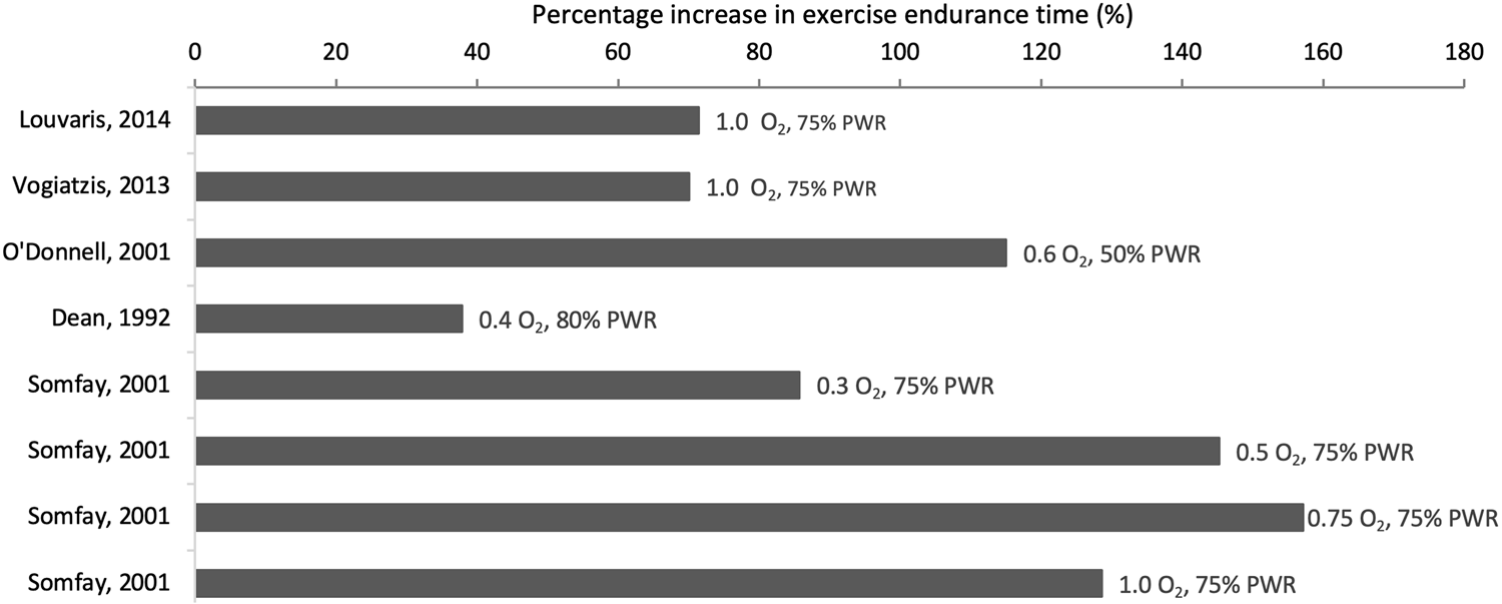
Percentage improvement in submaximal exercise endurance time during hyperoxia compared to normoxia in patients with COPD. Fractions of inspired oxygen concentration (FIO_2_) and peak work rate (PWR) are shown for each study. The study by Somfay et al. 2001, shows data from the same patients exercising at different FIO_2_

Patients with Chronic Obstructive Pulmonary Disease (COPD) exhibit limited endurance time compared to healthy age-matched individuals [1]. This is due to a combination of factors—ventilatory constraints, arterial hypoxemia from ventilation/perfusion inequality, reduced systemic oxygen delivery and peripheral muscle dysfunction [1]. During exercise, as ventilatory demand increases, patients with COPD exhibit limited ventilatory capacity due to expiratory flow limitation and dynamic hyperinflation, leading to a mismatch between ventilatory capacity and demand, which intensifies dyspnoea [1, 2]. Moreover, oxygen delivery to the respiratory and locomotor muscles becomes insufficient because of the limited increase in cardiac output and the arterial hypoxemia, which can worsen with increasing exercise intensity [1, 3]. In addition to the limited oxygen availability to the working muscles, locomotor muscle fibre abnormalities accelerate the onset of muscle fatigue leading to increased peripheral muscle discomfort. Oxygen supplementation is known to enhance exercise endurance time at a fixed power output in patients with COPD [4–8] and is often applied to prolong endurance time during pulmonary rehabilitation. Studies in COPD investigating endurance time and physiological variables during exercise at a fixed power output during hyperoxia compared to normoxia show reduced ventilatory drive and metabolic load, and improved dynamic ventilatory mechanics, which together result in a delay in the attainment of critical ventilatory constraints and the attendant intolerable respiratory discomfort [6–8]. In the study by Somfay and colleagues improvements in endurance time were dose-dependent according to different fractions of inspired oxygen (FIO_2_), thereby showing optimal effects of hyperoxia at 0.5 FIO_2_ without any further improvements at 0.75 and 1.0 FIO_2_ [6]. Effects of hyperoxia on endurance time from relevant studies [4–8] are provided in Fig. 1. However, these studies principally [6–8] focused on ventilatory variables leading to conclusions primarily about the magnitude of improvement in ventilatory mechanics during exercise in hyperoxia compared to normoxia. In the present report of our studies we have taken the unique and broad set of central hemodynamic, ventilatory and cerebrovascular, respiratory and locomotor muscle oxygen delivery measurements to reason through why endurance is elevated by hyperoxia and in the end what terminates exercise endurance as factor(s) common to normoxia and hyperoxia. To this end, the current review paper analyses findings from experiments investigating the effect of oxygen supplementation on cerebrovascular, systemic (i.e., whole body), respiratory and locomotor muscle oxygen availability on the same individuals with advanced COPD (Table 1) exercising to the limit of tolerance in normoxia and normobaric hyperoxia (1.0 FIO_2_). Data presented here have been extracted from our group's studies [4, 5]. To this end the current review paper analyses findings from experiments investigating the effect of oxygen supplementation on cerebrovascular, systemic (i.e., whole body), respiratory and locomotor muscle oxygen availability on the same individuals with advanced COPD (Table 1) exercising to the limit of tolerance in normoxia and normobaric hyperoxia (100% oxygen). Data presented here have been extracted from our group's studies [4, 5].

**Table 1:** Lung function and demographic phenotyping of COPD patients

Variables	Value
Age (years)	66 (5)
Height (cm)	169 (9)
Weight (kg)	73 (14)
BMI (kg/m^2^)	25.5 (4.1)
FFMI (kg/m^2^)	17.7 (2.6)
FEV_1_ (L)	1.2 (0.4)
FEV_1_ (% pred)	42.2 (12.9)
FVC (L)	2.7 (0.7)
FVC (% pred)	71.2 (17.2)
TLC (L)	8.1 (1.8)
TLC (% pred)	130 (20)
RV (L)	4.4 (1.3)
RV (% pred)	209 (50)
FRC (L)	5.5 (1.3)
FRC (% pred)	171 (30)
D_L, CO_ (% pred)	37.4 (11.1)
PaO_2_ (mmHg)	71 (10)
PaCO_2_ (mmHg)	39 (7)
SaO_2_ (%)	93 (4)
pH	7.44 (0.29)

Data are presented as means (SD) for *n* = 12 [5]. *BMI* body mass index, *FFMI* fat-free mass index, *FEV*_*1*_ forced expiratory volume in the first second, *% pred* % predicted, *FVC* forced vital capacity, *TLC* total lung capacity, *RV* residual volume, *FRC* functional residual capacity, *D*_*L, CO*_ diffusing capacity of the lung for carbon monoxide, *PaO*_*2*_ arterial oxygen tension, *PaCO*_*2*_ arterial carbon dioxide tension, *SaO*_*2*_ arterial oxygen saturation

The focus of this article is on two issues: The first is “isotime” (which is the time at which patients became exhausted in normoxia), where it is helpful to compare physiological variables in normoxia and hyperoxia to identify those which remain more favourable to exercise in hyperoxia, thus likely facilitating increased endurance. The second is to compare physiological variables at exhaustion under both FIO_2_ conditions to identify those that, while more favourable at isotime, have reached similar values at exhaustion. Such variables likely indicate critical factors determining endurance time.

Thus, the first aim of the present review is to identify the mechanisms of improved endurance time in hyperoxia, and secondly, to identify which physiological factors determine exhaustion in both hyperoxia and normoxia.

## Analysis

Experiments were conducted in two visits. In visit 1, patients underwent an incremental exercise test to the limit of tolerance [peak work rate (WRpeak)] while breathing room air. In visit 2, patients undertook two constant-load exercise tests at the same work rate (75% WRpeak) while breathing room air first and 100% oxygen afterwards. 100% oxygen was administered by having subjects inspire from a Douglas bag that was connected to the inspiratory port of a non-re-breathing two-way valve by wide-bore tubing. The same apparatus was utilised during room air breathing to ensure that patients were blinded to the inspired gas mixture each time. During each constant-load exercise test, recordings of ventilatory variables were performed breath-by-breath. During exercise, intercostal, abdominal, and quadriceps muscle blood flow was assessed by near-infrared spectroscopy (NIRS) using the light-absorbing tracer indocyanine green (ICG) dye as indicator [9]. Local muscle blood flow was assessed via three pairs of NIRS optodes that were placed on the skin over (1) the left seventh intercostal space at the midaxillary line, (2) the upper rectus abdominis, and (3) the left vastus lateralis muscle 10–12 cm above the knee, respectively. Cerebral cortex blood flow was assessed by one pair of NIRS optodes that was placed on the skin over the left frontal cortex region of the forehead. The respective oxygen delivery was calculated as the product of the appropriate blood flow and arterial oxygen content. Cardiac output was measured by the standard dye dilution method using ICG [10]. Arterial blood pressure was measured invasively through the right radial artery using a Nihon Kohden monitor. Arterial blood gases were measured at rest and exhaustion during the final minute of each test; percentage arterial oxygen saturation (%SaO_2_), arterial tensions of O_2_ (PaO_2_) and CO_2_ (PaCO_2_), Hb concentration, and lactate concentration were measured from 2-ml blood samples using a blood gas analyser combined with a co-oximeter. As endurance time was expected to be significantly prolonged while subjects breathed pure oxygen, measurements during these trials were also performed at the time point where exercise in room air was terminated (i.e., at isotime). Detailed methods can be found in the previous studies from our group [4, 5].

### Cerebrovascular oxygen availability and brain oxygenation (Fig. 2)

**Fig. 2:**
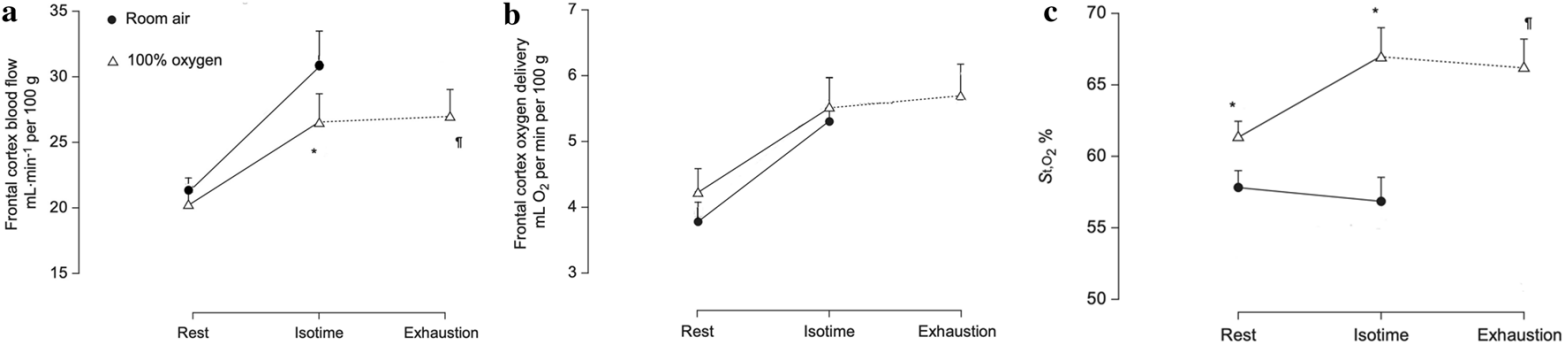
Frontal cortex cerebrovascular oxygenation and haemodynamic responses: (a) blood flow, (b) oxygen delivery and (c) fractional oxygen saturation (St,O_2_%) at rest and at exhaustion while breathing room air (closed symbols), or 100% oxygen (open symbols). Values are means (SEM) for *n* = 12 [5]. *p < 0.05 compared with room air at the same time point of exercise; ¶*p* < 0.05 compared with exhaustion in room air

In patients with COPD prolonged exercise endurance time with oxygen supplementation has been attributed to the alleviation of ventilatory constraints, improved oxygen delivery to the locomotor muscles and reduced perceptions of dyspnoea and leg discomfort [11–15]. Whether improvement in endurance time in these patients is partly due to increased brain oxygen availability remained until recently unexplored. Increased brain oxygen availability could alter symptom perception and/or cardio-pulmonary responses to exercise, both of which could affect endurance time.

At both exercise isotime and exhaustion, frontal cortex cerebral blood flow was lower in hyperoxia compared to normoxia (Fig. 2a) most likely due to arteriolar vasoconstriction with high oxygen supplementation [16]. However, cerebral oxygen delivery was not different between normoxia and hyperoxia at either isotime or exhaustion (Fig. 2b), due to higher arterial oxygen concentrations. Thus, improved exercise endurance time during hyperoxia was not attributed to increased cerebrovascular oxygen availability [5]. Interestingly, frontal cortex oxygen saturation measured by NIRS (StO_2_) was higher in hyperoxia (Fig. 2c) despite similar oxygen delivery, presenting the possibility that the brain might have been better oxygenated despite lower blood flow and that this could have contributed to prolonged endurance time in hyperoxia. However, at exhaustion in hyperoxia, frontal cortex oxygen StO_2_ remained similar to values at isotime, considerably exceeding those at exhaustion in normoxia, thus suggesting that brain oxygenation is not responsible for the patient’s decision to stop exercise. The major caveat with this analysis is that frontal cortex parameters may not reflect those of the regions of the brain responsible for those decisions. Similar minute ventilation at isotime despite higher arterial PaCO_2_ and lower dyspnoea scores (Table 2) imply lower respiratory drive in hyperoxia [17, 18], thereby suggesting there might be a role for the brain in the different physiological responses and endurance times between normoxia and hyperoxia.

**Table 2:** Breathing pattern and gas exchange in normoxia and hyperoxia during exercise at 75% of WRpeak

Variables	Air exhaustion	100% oxygen isotime	100% oxygen exhaustion
Time, s	406 (36)		696 (48)*
V_E_, l/min	35.1 (3.0)	33.6 (2.5)	39.6 (2.6)*
V_T_, l	1.23 (0.09)	1.30 (0.12)*	1.34 (0.13)*
f, breaths/min	29.0 (2.0)	26.0 (2.0)*	29.0 (2.1)
Ti/Ttot. %	35.0 (2.0)	45.6 (2.7)*	44.1 (2.6)*
ΔIC, l	− 0.196 (0.03)	− 0.110 (0.02)	− 0.119 (0.03)
SaO_2_, %	88 (2)	99 (1)*	99 (1)*
PaO_2_, mmHg	67 (4)	540 (14)*	533 (23)*
PaCO_2_, mmHg	48 (2)	51 (3)*	52 (3)*
Lactate concentration (mmol/l)	4.7 (0.5)	3.2 (0.5)*	3.2 (0.6)*
Borg dyspnoea scores	7.4 (2.0)	4.9 (1.0)*	6.3 (1.3)
Borg leg discomfort scores	5.9 (2.1)	4.6 (1.7)*	5.9 (2.0)

Values are expressed as mean (SEM) for *n* = 10 [4, 5]. *WRpeak* peak work rate in room air, *Ti/Tot* duty cycle of inspiration, *ΔIC* change in inspiratory capacity from baseline; *p < 0.05 versus exhaustion in room air.

### Ventilatory capacity versus ventilatory demand (Table 2, Fig. 3).

**Fig. 3:**
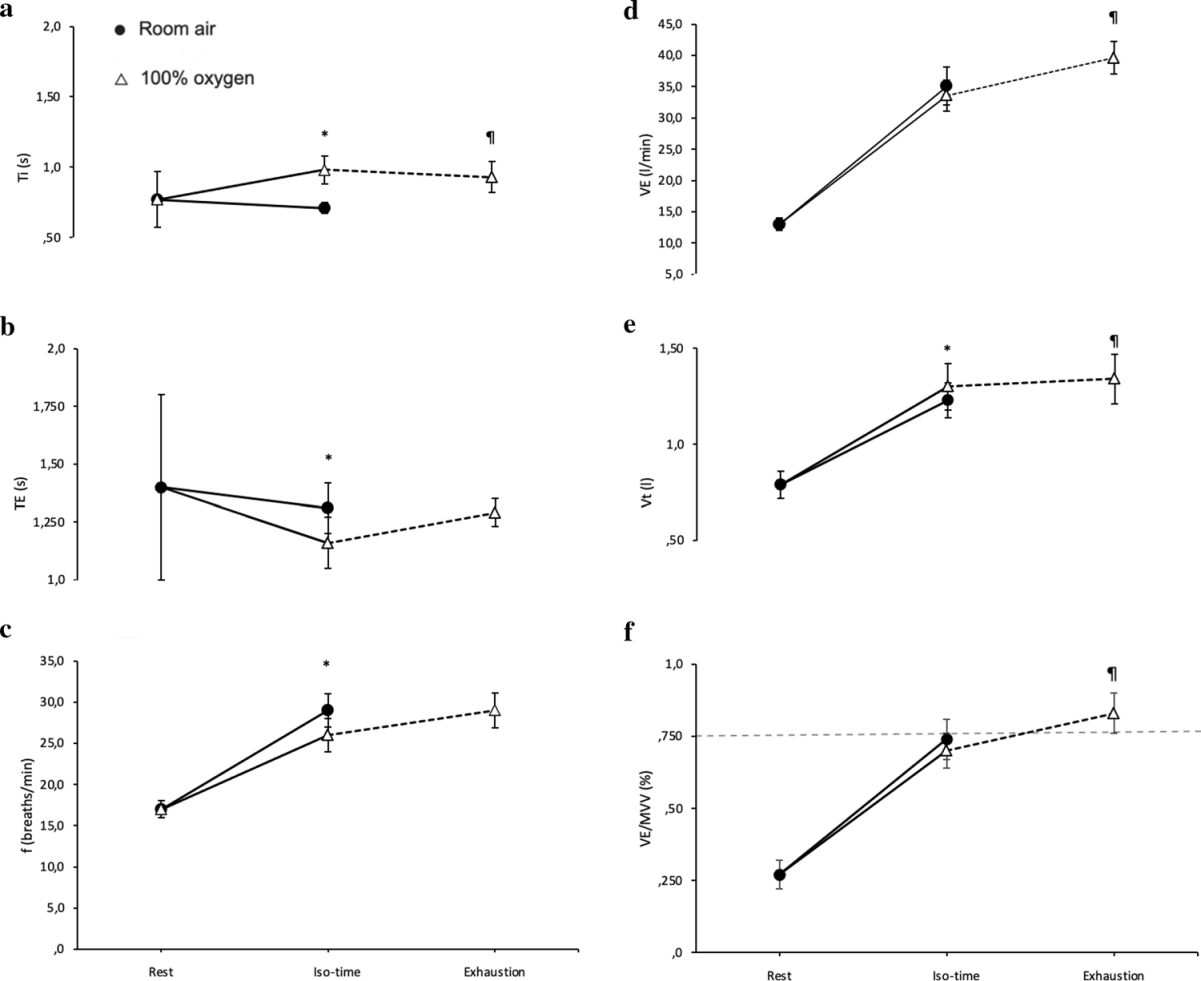
Breathing pattern responses: (a) time of inspiration (T_I_), (b) time of expiration (T_E_), (c) breathing frequency (f), (d) minute ventilation (V_E_), (e) tidal volume (V_T_), (f) breathing reserve (V_E_/MVV), all recorded at rest, at isotime and at exhaustion while breathing room air (closed symbols) or 100% oxygen (open symbols). Values are means (SEM) for *n* = 10 [4, 5]. Isotime data are those obtained on 100% oxygen at the same time as at exhaustion on room air. *p < 0.05 compared with room air *at the same time point of exercise*; ¶*p* < 0.05 compared with exhaustion in room air. Significant differences between data at isotime or exhaustion and rest are not indicated on the figure. Dotted line on Fig. 3f denotes the level of critical ventilatory constraints

Ventilation was similar at isotime in normoxia and hyperoxia but rose to above critically sustainable values at exhaustion [4, 5]. Respiratory frequency in hyperoxia, lower at isotime, increased with similar values observed at exhaustion in both conditions. Arterial PaCO_2_ was higher in hyperoxia, but without difference between isotime and exhaustion. Arterial lactate, lower at isotime in hyperoxia compared to normoxia, remained lower at exhaustion in hyperoxia. The isotime data suggest that hyperoxia lowered respiratory drive and the exhaustion comparison shows hyperoxia facilitated greater ventilation without greater dyspnoea. Taken together, the data suggest a key role for ventilatory responses in determining endurance time, with hyperoxia reducing drive to breathe and thus delaying symptoms.

Our COPD population exhibited exercise-induced dynamic hyperinflation as reflected by the decrease in inspiratory capacity from baseline in normoxia, which was equal to 196 ml [19] (Table 2). Moreover, ventilatory constraints at exhaustion in normoxia, were manifested by V_E_/MVV reaching the critical point of ventilatory constraints (i.e.: 75% V_E_/MVV) (Fig. 3f). Besides the restrictive ventilatory mechanical constraints limiting endurance time in normoxia [2], a limited delivery of oxygen to the locomotor muscles, resulting in increased lactate (Table 2), may have constituted an additional reason for limiting exercise tolerance in normoxia.

In hyperoxia V_E_ was lower than in normoxia at isotime. However, at exhaustion in hyperoxia, V_E_ was greater than in normoxia most likely reflecting the increase in exercise time in this condition. In addition, an FIO_2_ of 1.0 lead to hypercapnia at exercise isotime in hyperoxia and thus, respiratory drive further increased from isotime to exhaustion in hyperoxia leading to increased ventilation and attainment of mechanical ventilatory constraints. During hyperoxia, patients did not show substantial dynamic hyperinflation neither at isotime nor at exhaustion (decrease in IC from baseline of 110–120 ml), which might partially explain the wider V_T_ expansion in hyperoxia compared to normoxia (Table 2). This was possibly due to reduced expiratory flow limitation that was reflected by the reduced expiratory time (T_E_) (Fig. 3b) and the increased Ti/ToT (Table 2) at both isotime and exhaustion in hyperoxia compared to exhaustion in normoxia. A possible mechanism for the reduced dynamic hyperinflation in hyperoxia has been reasoned to be associated with the altered breathing pattern during hyperoxia compared to normoxia (i.e., alterations in respiratory timing, increased expiratory flow and thus enhanced lung emptying) [7].

### Circulatory effects of breathing 100% oxygen during exercise (Fig. 4)

**Fig. 4:**
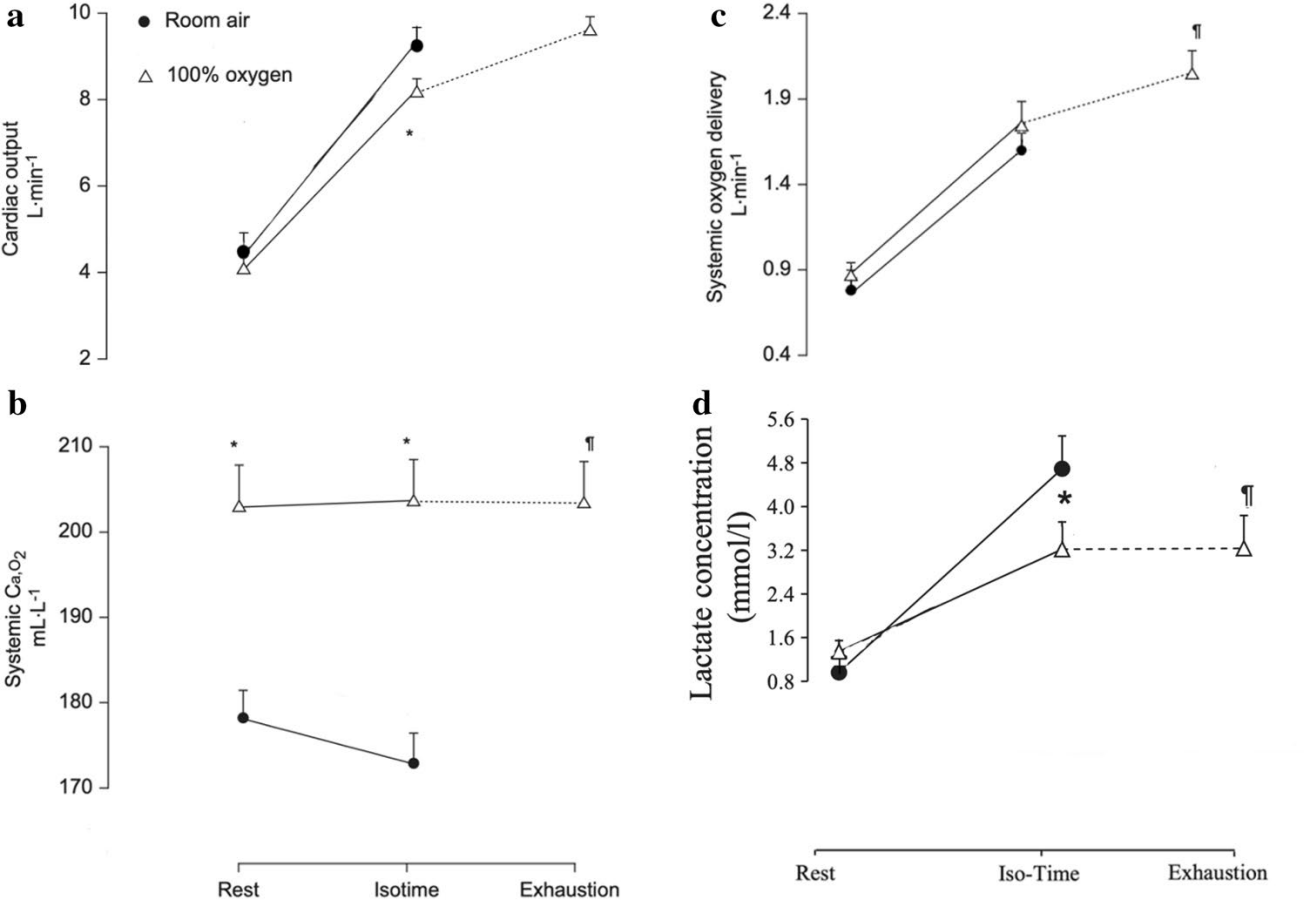
Central haemodynamic responses: (a) cardiac output, (b) systemic arterial oxygen content (Ca,O_2_), (c) systemic oxygen delivery, (d) lactate concentration, recorded at rest, at isotime and at exhaustion while breathing room air (closed symbols) or 100% oxygen (open symbols). Values are means (SEM) for *n* = 10 [4, 5]. Isotime data are those obtained on 100% oxygen at the same time as at exhaustion on room air. *p < 0.05 compared with room air *at the same time point of exercise*; ¶*p* < 0.05 compared with exhaustion in room air. Significant differences between data at isotime or exhaustion and rest are not indicated here

At isotime during hyperoxia, cardiac output was approximately 10% lower than in normoxia (Fig. 4a), possibly because of lower sympathetic stimulation resulting from abolition of arterial hypoxemia. However, cardiac output reached the same values at exhaustion under both conditions. With exercise stopping at the same cardiac output values in normoxia and hyperoxia, this makes cardiac function a candidate factor in determining endurance time: when cardiac output can no longer be increased, further exercise is curtailed. In hyperoxia, this took longer than in normoxia, possibly because of lower sympathetic stimulation in hyperoxia [20].

During hyperoxia, the expectedly higher arterial oxygen content was seen (Fig. 4b), which resulted in higher systemic oxygen delivery despite the lower cardiac output. Interestingly, at exhaustion in hyperoxia, systemic oxygen delivery was significantly higher than at exhaustion in normoxia. This finding does not support global oxygen transport as a factor determining endurance time. Of course, global oxygen transport data do not reveal how much oxygen was available to key tissues important to endurance time such as the locomotor, respiratory or cardiac muscles or the relevant parts of the brain.

### Respiratory and locomotor muscle blood flow and oxygen delivery (Fig. 5)

**Fig. 5:**
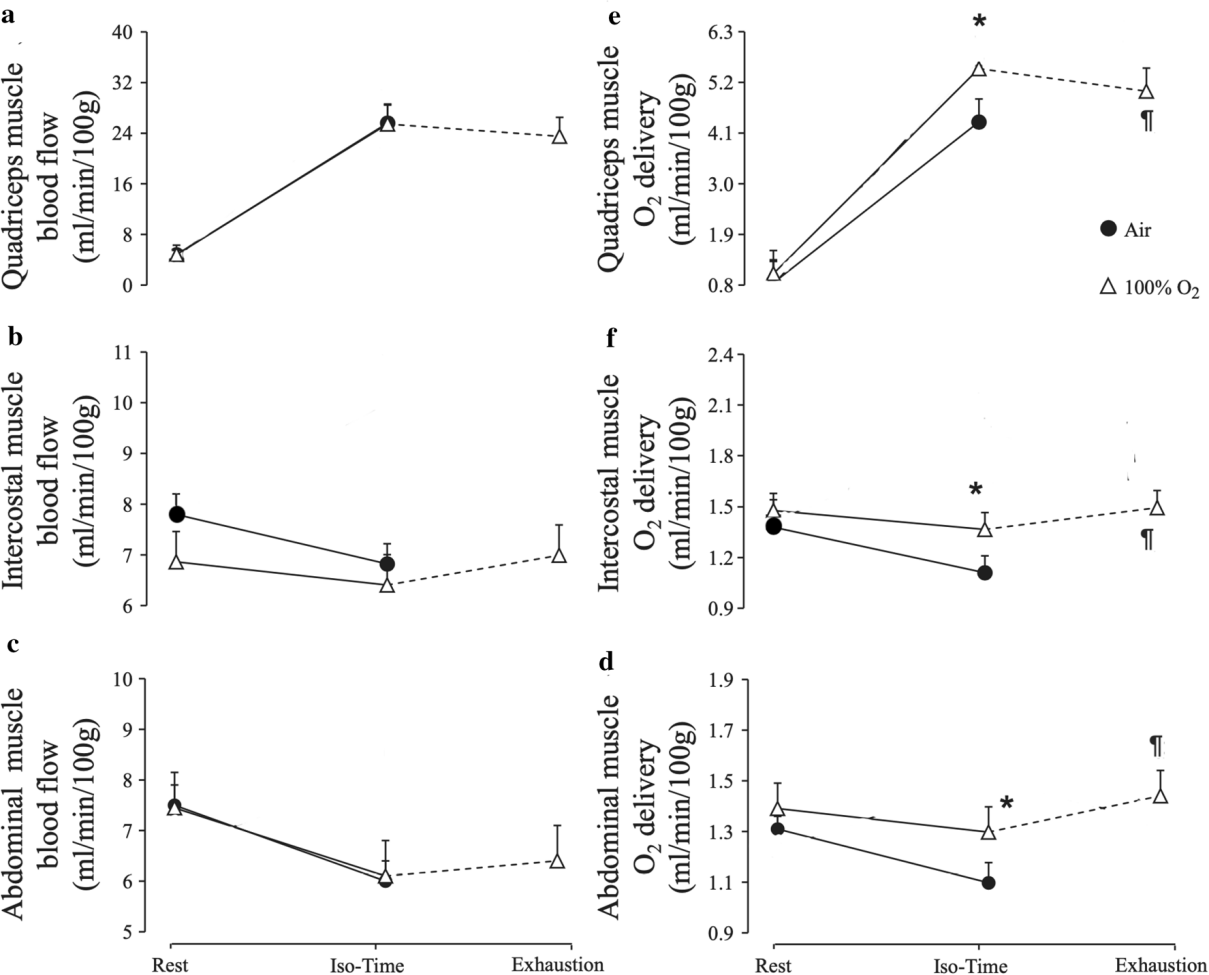
Quadriceps and respiratory muscle haemodynamic responses: (**a**) quadriceps muscle blood flow; (**b**) intercostal muscle blood flow; (**c**) abdominal muscle blood flow; (**d**) quadriceps muscle oxygen delivery; (**e**) intercostal muscle oxygen delivery; (**f**) abdominal muscle oxygen delivery recorded at rest, at the time of exhaustion in room air (isotime), and at exhaustion while subjects breathed 100% oxygen (open symbols), or room air (closed symbols). Values are means (SEM) for *n* = 10 [4]. Isotime data are those obtained on 100% oxygen at the same time as at exhaustion on room air. *p < 0.05 compared with room air at the same time point of exercise; ¶*p* < 0.05 compared with exhaustion in room air

Intercostal, abdominal and locomotor muscle blood flow (all assessed by NIRS using ICG) were not different between normoxia and hyperoxia at isotime or at exhaustion (Fig. 5) [4]. However, oxygen delivery, both to these respiratory muscles and the locomotor muscles, was significantly greater during hyperoxia compared to normoxia at both isotime and exhaustion [4]. Note that diaphragm blood flow cannot be measured by NIRS because its probes, placed on the skin surface, cannot access signals from that muscle because of anatomical distance from the skin.

This leads to the notion that a principal enabler of longer endurance time during hyperoxia is elevated oxygen delivery to the respiratory and locomotor muscles. The increase in oxygen delivery was associated with reduced dyspnoea and leg discomfort at exercise isotime (Table 2) and lower lactate levels. In fact Louvaris et al. [21] have shown that improved intercostal and abdominal muscle oxygen delivery is associated with reduced dyspnoea at exercise isotime.

However, the finding that locomotor and respiratory muscle oxygen availability remained greater at exhaustion in hyperoxia compared to normoxia, does not support a role for these factors in determining the actual time to exhaustion.

## Proposed mechanisms of prolonged endurance time during hyperoxia in COPD

Discussion of why hyperoxia, compared to normoxia, significantly increased endurance time (by 5 min, approximately a factor of two) [4, 5] during exercise sustained at 75% PWR is of great interest, and the data presented above allow some insight into the responsible physiological mechanisms. Two separate but related questions need to be addressed: First, how hyperoxia enables longer endurance times and second, what factor(s) determine exhaustion—the point at which the patient can no longer continue exercise. The latter question should be answered with mechanisms that explain exhaustion under both conditions.

### How hyperoxia prolongs endurance time at a given power output

The logic underlying analysis of this question is that at isotime (the time during hyperoxic exercise corresponding to the point of exhaustion during normoxic exercise), values of physiological variables of importance to endurance should be more favourable in hyperoxia compared to their values at exhaustion in normoxia.

First, respiratory drive is reduced by hyperoxia. While this allows arterial PaCO_2_ to rise, the abolition of arterial hypoxemia likely results in less carotid body neural signalling to the respiratory centres [18]. This in turn reduces minute ventilation, the work of breathing and associated dyspnoea, and thus delays the decision to stop exercise. Second, cardiac output is lower in hyperoxia, likely because of reduced sympathetic activation from elimination of arterial hypoxemia [20]. This leaves some reserve that can be used to enable continued exercise, which may require additional blood flow due to temperature increases or slow phase oxygen uptake increase. Oxygenation status of the brain is greater in hyperoxia (based on frontal cortex measurements). Better oxygenation of regions of the brain responsible for symptom perception and resulting decision-making by the patient may attenuate those symptoms and enable longer exercise time [22, 23]. Arterial PaO_2_ in hyperoxia exceeds 500 mmHg, which must increase intracellular PaO_2_. Oxidative phosphorylation, driven by intracellular PaO_2_, can, therefore, proceed faster, raising maximal oxygen utilization rate potential [24]. The same absolute oxygen consumption rate is now a lower fraction of a higher maximal oxygen consumption rate, enabling longer exercise time. Faster oxidative phosphorylation from the higher intracellular PaO_2_ also allows more of the products of glycolysis to flow into the citric acid cycle of the locomotor and respiratory muscles and not be diverted into lactate production [25]. Lower lactate levels likely reduce both dyspnoea and leg discomfort (less acidosis) and allow for longer exercise time.

### What are the factor(s), which determine exhaustion?

The logic we apply to probe this question is to compare physiological variables a) at exhaustion in normoxia with their values at the same time during hyperoxic exercise (isotime) at the same power output, and b) at exhaustion under both FIO_2_ conditions. We expect determining factors to exhibit a) more favourable values in hyperoxia than in normoxia at isotime but b) similar (or possibly even less favourable) values at exhaustion under both FIO_2_ conditions.

The physiological variables that fit the pattern expected for a limiting factor (i.e., favourable in hyperoxia at isotime but not significantly different at exhaustion) were cardiac output (Fig. 4a), breathing frequency, expiratory time (Table 2) and ventilation (Fig. 3). However, respiratory muscle and locomotor muscle oxygen delivery and frontal cortex tissue oxygenation levels were all higher at exhaustion in hyperoxia than in normoxia, and arterial lactate was lower.

At exhaustion in hyperoxia, cardiac output reached the same absolute value as in normoxia indicating limited central haemodynamic response. Consequently, at exhaustion in both normoxia and hyperoxia cardiac output exceeded 95% of peak cardiac output previously reported by our group for patients with comparable COPD severity during exercise in room air [26]. This finding indicates, limited cardiovascular reserve.

Likewise, expiratory time reached the same values at exhaustion in normoxia and hyperoxia, thus indicating prolonged expiratory phase most likely secondary to expiratory flow limitation (EFL). EFL is known to intensify dyspnoea [27]. Furthermore, breathing frequency was identical at exhaustion in both conditions. However, tidal volume was still greater at exhaustion in hyperoxia compared to exhaustion in normoxia, thereby causing minute ventilation at exhaustion in hyperoxia to exceed the critical level of ventilatory constraints (i.e.: V_E_/MVV > 75–80%) that is indicative of ventilatory limitation [28]. When V_E_/MVV reaches or exceeds 75–80% in room air, breathing discomfort is intensified and is associated with exercise limitation in COPD [28]. The study by O’Donnell et al. (2001) showed that endurance time during cycling sustained at 75% of peak work capacity was limited when V_E_/MVV reached 83% in normoxia and 84% in hyperoxia [7]. Our studies [4, 5] and this by O’Donnell et al. (2001) show that dyspnoea at exhaustion in hyperoxia was not different compared to that in normoxia (Table 2).

Therefore, according to this analysis, the likely major reasons for terminating exercise in both hyperoxia and normoxia were having reached central cardiovascular and ventilatory limitation, which took longer during hyperoxia than in normoxia due to greater oxygen availability. These findings, in turn, highlight the value of exercise in hyperoxic conditions, which delays the increase in cardiac output and the critical level of ventilatory limitation (both due to increased arterial oxygenation).

#### Likelihood of blood flow redistribution from the respiratory to the locomotor muscles

Whilst oxygen supplementation (hyperoxia) primarily enhances arterial oxygen concentration, it also lessens the work of breathing [6, 29–31]. Decreased work of breathing has been shown to be associated with less ‘respiratory muscle metaboreflex’ triggering, causing less sympathetic efferent discharge to the leg muscles, thus allowing improved leg muscle blood flow and oxygen delivery [29, 32, 33]. However, whether the increase in endurance time in COPD occurs on the basis of improved blood flow to the locomotor muscles remained largely unknown for some time. Whilst previous reports suggested that reducing the work of breathing was associated with an increase in locomotor muscle blood flow [32, 33], the findings from our studies do not support this hypothesis as respiratory and locomotor muscle blood flow were not different between normoxia and hyperoxia at neither exercise isotime nor at exhaustion (Fig. 5) [4].

**Fig. 6:**
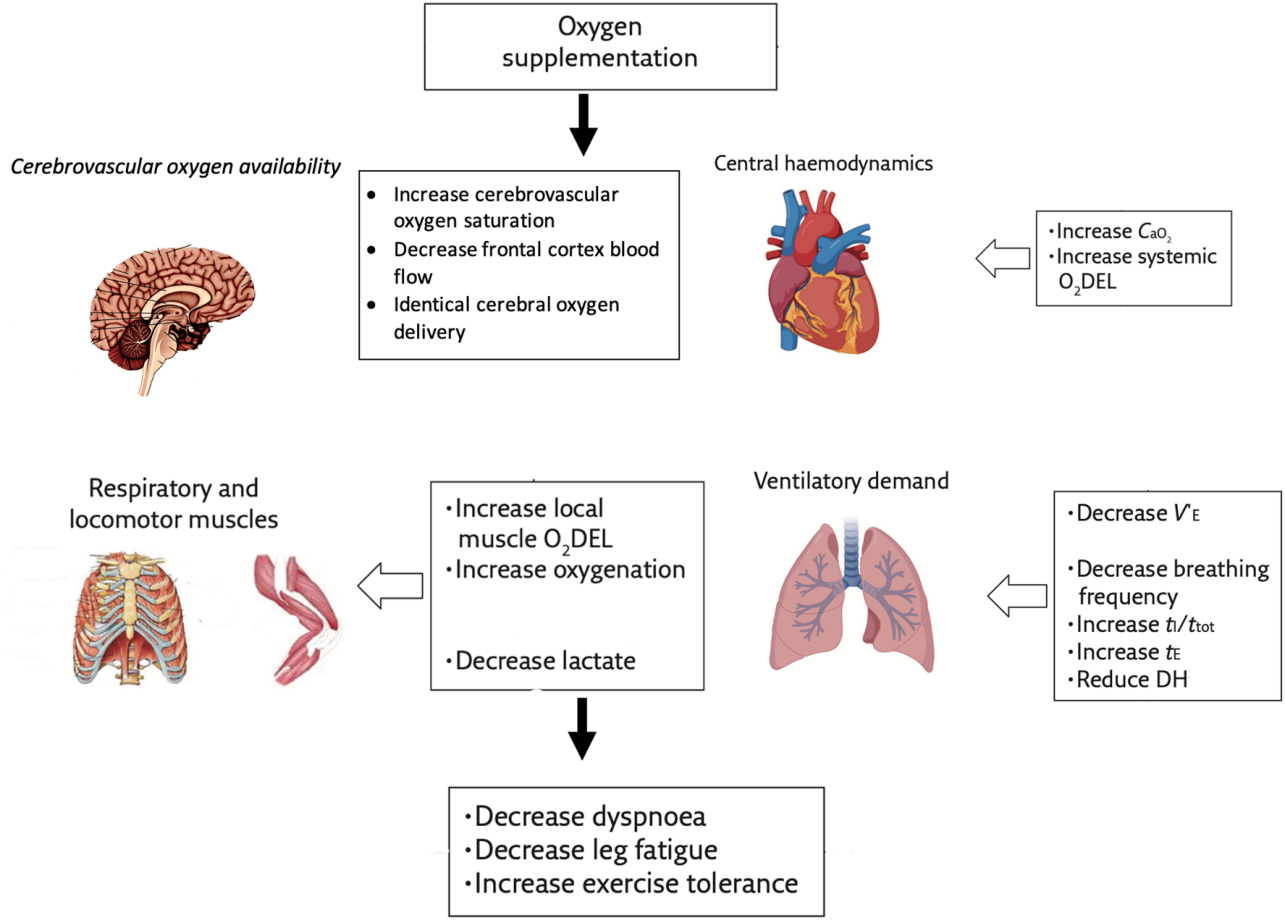
A schematic representation conceptualising the acute physiological responses of patients with COPD during exercise breathing oxygen compared with exercise in room air at isotime (when work completed is the same between normoxia and hyperoxia). *t*_*E*_ expiratory time, *DH* dynamic hyperinflation, *CO* cardiac output, *O*_*2*_*DEL* oxygen delivery, *SVC* systemic vascular conductance

#### Comparison of our studies with the literature

Previous studies have shown that compared to exercise in room air, oxygen supplementation prolongs exercise endurance time by 40–160% relative to normoxia. In our studies [4, 5] endurance time was increased by 75% compared to normoxia, which is within the range previously reported. Comparing the findings of our study to previously published studies [6–8], hyperoxia elicited an identical pattern in delaying ventilatory constraints that is in line with our findings. Our report expands on the multifactorial effects of hyperoxia, besides reducing the stress on the ventilatory and cardiovascular systems, the reliance on anaerobic glycolysis and the perception of symptom intensity, by showing improved cerebral oxygen saturation and oxygen delivery to locomotor and respiratory muscles at exercise isotime with normoxia.

In healthy individuals, the inability of the circulatory system to meet the requirements of locomotor and respiratory muscles during normoxic heavy exercise contributes to the termination of exercise before reaching critical ventilatory constraints as opposed to COPD patients which exhibit both circulatory and ventilatory constraints [34].

## Conclusions

Several factors improve endurance time during hyperoxia in patients with COPD as follows (Fig. 6). During hyperoxia, respiratory drive is reduced, lessening dyspnoea. Moreover, cardiac output is lower, leaving reserve in blood flow. Oxygenation status of the brain is greater, perhaps reducing symptom perception. Oxygen delivery to the respiratory and locomotor muscles is higher, increasing intracellular PaO_2_ and thus enhancing oxidative phosphorylation. Finally, blood lactate is reduced, lowering acidosis, dyspnoea and leg discomfort. Time to exhaustion on the other hand appears to be largely determined by having reached both ventilatory and cardiac limits of function.

### Clinical implications

Exercise training in hyperoxic conditions during pulmonary rehabilitation allows greater exercise tolerance at a given exercise intensity or sustaining higher exercise intensities for a given period of time, thus potentially inducing greater physiological adaptations compared to exercise training in normoxia. Furthermore, regular exercise training with elevated FIO_2_ likely raises mitochondrial PO_2_, which might reduce hypoxia-related adaptive gene expression and counter muscle adaptive changes.

## References

[1] Vogiatzis I, Zakynthinos S (2012). Factors limiting exercise tolerance in chronic lung diseases. Compr Physiol.

[2] Calverley PMA, Koulouris NG (2005). Flow limitation and dynamic hyperinflation: key concepts in modern respiratory physiology. Eur Respir J.

[3] Vogiatzis I (2010). Intercostal muscle blood flow limitation during exercise in chronic obstructive pulmonary disease. Am J Respir Crit Care Med.

[4] Louvaris Z (2014). Blood flow does not redistribute from respiratory to leg muscles during exercise breathing heliox or oxygen in COPD. J Appl Physiol (1985).

[5] Vogiatzis I (2013). Cerebral cortex oxygen delivery and exercise limitation in patients with COPD. Eur Respir J.

[6] Somfay A (2001). Dose-response effect of oxygen on hyperinflation and exercise endurance in nonhypoxaemic COPD patients. Eur Respir J.

[7] O'Donnell DE, D'Arsigny C, Webb KA (2001). Effects of hyperoxia on ventilatory limitation during exercise in advanced chronic obstructive pulmonary disease. Am J Respir Crit Care Med.

[8] Dean NC (1992). Oxygen may improve dyspnea and endurance in patients with chronic obstructive pulmonary disease and only mild hypoxemia. Am Rev Respir Dis.

[9] Boushel R (2000). Regional blood flow during exercise in humans measured by near-infrared spectroscopy and indocyanine green. J Appl Physiol (1985).

[10] Dow P (1956). Estimations of cardiac output and central blood volume by dye dilution. Physiol Rev.

[11] Gallagher CG (1994). Exercise limitation and clinical exercise testing in chronic obstructive pulmonary disease. Clin Chest Med.

[12] O'Donnell DE, Webb KA (2008). The major limitation to exercise performance in COPD is dynamic hyperinflation. J Appl Physiol (1985).

[13] Barbera JA (1991). Gas exchange during exercise in mild chronic obstructive pulmonary disease. Correlation with lung structure. Am Rev Respir Dis.

[14] Mahler DA (1984). Right ventricular performance and central circulatory hemodynamics during upright exercise in patients with chronic obstructive pulmonary disease. Am Rev Respir Dis.

[15] Debigaré R, Maltais F (2008). The major limitation to exercise performance in COPD is lower limb muscle dysfunction. J Appl Physiol (1985).

[16] Cornet AD (2013). The potential harm of oxygen therapy in medical emergencies. Critical Care (London, England).

[17] Palange P (2005). Supplemental oxygen and heliox: ‘new’ tools for exercise training in chronic obstructive pulmonary disease. Curr Opin Pulm Med.

[18] Casaburi R (1980). Alteration by hyperoxia of ventilatory dynamics during sinusoidal work. J Appl Physiol Respir Environ Exerc Physiol.

[19] O'Donnell DE, Revill SM, Webb KA (2001). Dynamic hyperinflation and exercise intolerance in chronic obstructive pulmonary disease. Am J Respir Crit Care Med.

[20] Marshall JM (1994). Peripheral chemoreceptors and cardiovascular regulation. Physiol Rev.

[21] Louvaris Z (2017). Improvement in respiratory muscle O 2 delivery is associated with less dyspnoea during exercise in COPD: Respiratory muscle O 2 delivery & dyspnea in COPD. Clin Respir J.

[22] Subudhi AW (2009). Frontal and motor cortex oxygenation during maximal exercise in normoxia and hypoxia. J Appl Physiol (1985).

[23] Imray CH (2005). Effect of exercise on cerebral perfusion in humans at high altitude. J Appl Physiol (1985).

[24] Wilson DF, Harrison DK, Vinogradov SA (2012). Oxygen, pH, and mitochondrial oxidative phosphorylation. J Appl Physiol.

[25] Fuhrmann DC, Brüne B (2017). Mitochondrial composition and function under the control of hypoxia. Redox Biol.

[26] Louvaris Z (2019). Cardiac output measurement during exercise in COPD: a comparison of dye dilution and impedance cardiography. Clin Respir J.

[27] Díaz O (2001). Breathing pattern and gas exchange at peak exercise in COPD patients with and without tidal flow limitation at rest. Eur Respir J.

[28] Neder JA (2018). Introduction: CPET in clinical practice. recent advances, current challenges and future directions. ERS Monograph.

[29] Amann M (2010). Impact of pulmonary system limitations on locomotor muscle fatigue in patients with COPD. Am J Physiol Regul Integr Comp Physiol.

[30] Bye PT (1985). Ventilatory muscle function during exercise in air and oxygen in patients with chronic air-flow limitation. Am Rev Respir Dis.

[31] O'Donnell DE, Bain DJ, Webb KA (1997). Factors contributing to relief of exertional breathlessness during hyperoxia in chronic airflow limitation. Am J Respir Crit Care Med.

[32] Dempsey JA (2002). Respiratory influences on sympathetic vasomotor outflow in humans. Respir Physiol Neurobiol.

[33] Dempsey JA (2006). Consequences of exercise-induced respiratory muscle work. Respir Physiol Neurobiol.

[34] Vogiatzis I (2009). Intercostal muscle blood flow limitation in athletes during maximal exercise. J Physiol.

